# Supplementary material for: Diffuse X-ray scattering from correlated motions in a protein crystal
Source: Nat Commun. 2020 Mar 9;11:1271. doi: 10.1038/s41467-020-14933-6 (PMC7062842; doi:10.1038/s41467-020-14933-6)
Supplement: Supplementary file 1 — Supplementary Information [file 41467_2020_14933_MOESM1_ESM.pdf]

Supplementary Information

Diffuse X-ray Scattering from Correlated Motions in a Protein Crystal

Meisburger et al

Contents

Supplementary Figures **2**

Supplementary Tables **16**

Supplementary Notes **19**

Supplementary Methods **20**

1 General Theory **20**
- 1.1 Total scattering cross-section 20
- 1.2 Coherent scattering from crystals 21
- 1.3 Diffuse Patterson function 22
- 1.4 Scattering simulation 22

2 Experimental Corrections **22**
- 2.1 Geometric factors 23
- 2.2 Corrections to experimental data 24
- 2.3 Incoherent scattering and absolute intensity 24

3 Lattice Dynamics Simulation **25**
- 3.1 Equations of motion in a rigid-body coordinate system 25
- 3.2 Normal modes 26
- 3.3 Displacement covariances for thermally-excited vibrations 27
- 3.4 Diffuse scattering and the one-phonon approximation 27

Supplementary References **29**

Supplementary Figures

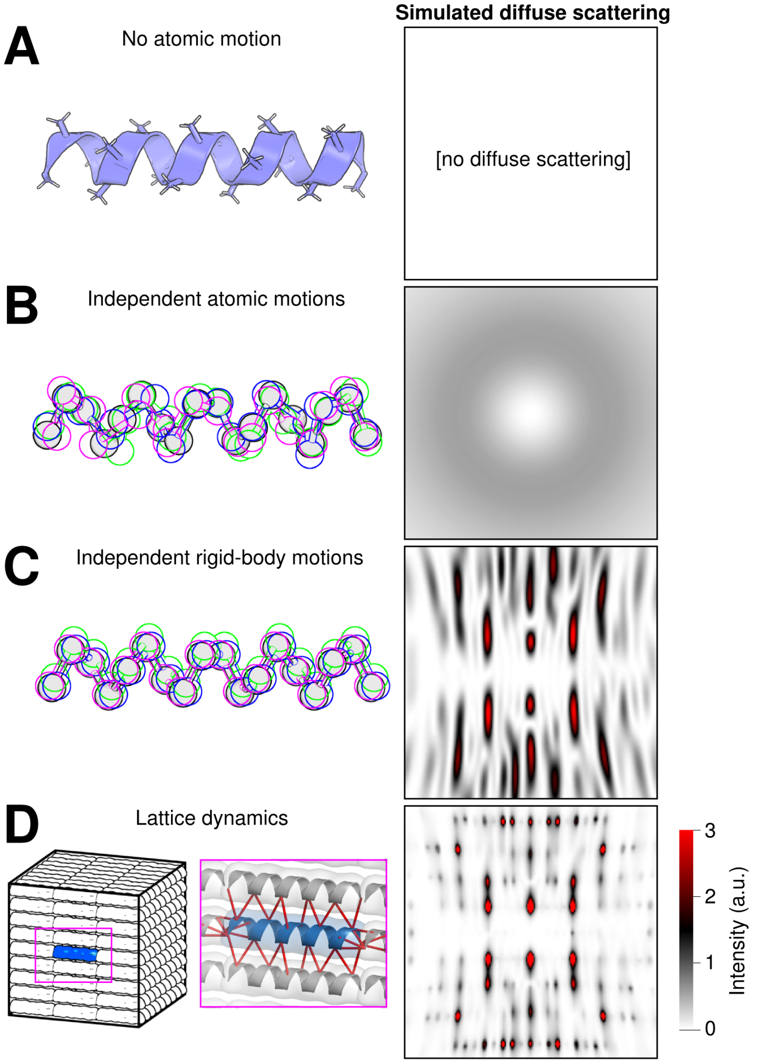

Supplementary Fig. 1: Diffuse scattering depends on how atomic displacements are correlated. To illustrate, scattering patterns were simulated for a crystal of an idealized alpha helix (panel A) with various types of motion. The simulated X-ray beam had a wavelength of 1 Å and was perpendicular to the helical axis (into the page). (A) If there is no atomic motion (left), then there is no diffuse scattering (right). (B) Uncorrelated atomic displacements with a B-factor of 20 Å^2. On the left, a representative ensemble is illustrated for C_α positions with the mean shaded in gray and three random samples outlined in different colors. The diffuse scattering is relatively featureless and isotropic (right). (C) Translational rigid body motion that is uncorrelated between unit cells. B-factors are the same as in panel B. The diffuse scattering (right) has oscillations related to the molecular transform [1]. (D) Helices move as in panel C except that motions are correlated between unit cells. The correlations were calculated from a vibrational lattice dynamics model, similar to the one developed for this study (see Methods), consisting of a periodic supercell (left) with a network of springs (red lines, inset). The potential energy function (Equation 5 in the Main Text) was chosen so that thermally excited vibrations at room temperature produced the same B-factors as in panels B-C. The simulated diffuse pattern (right) contains intense halo features that coincide with the Bragg peaks (not shown). Although the models in panels B-D have identical B-factors and cannot be distinguished by Bragg diffraction, they predict very different diffuse scattering.

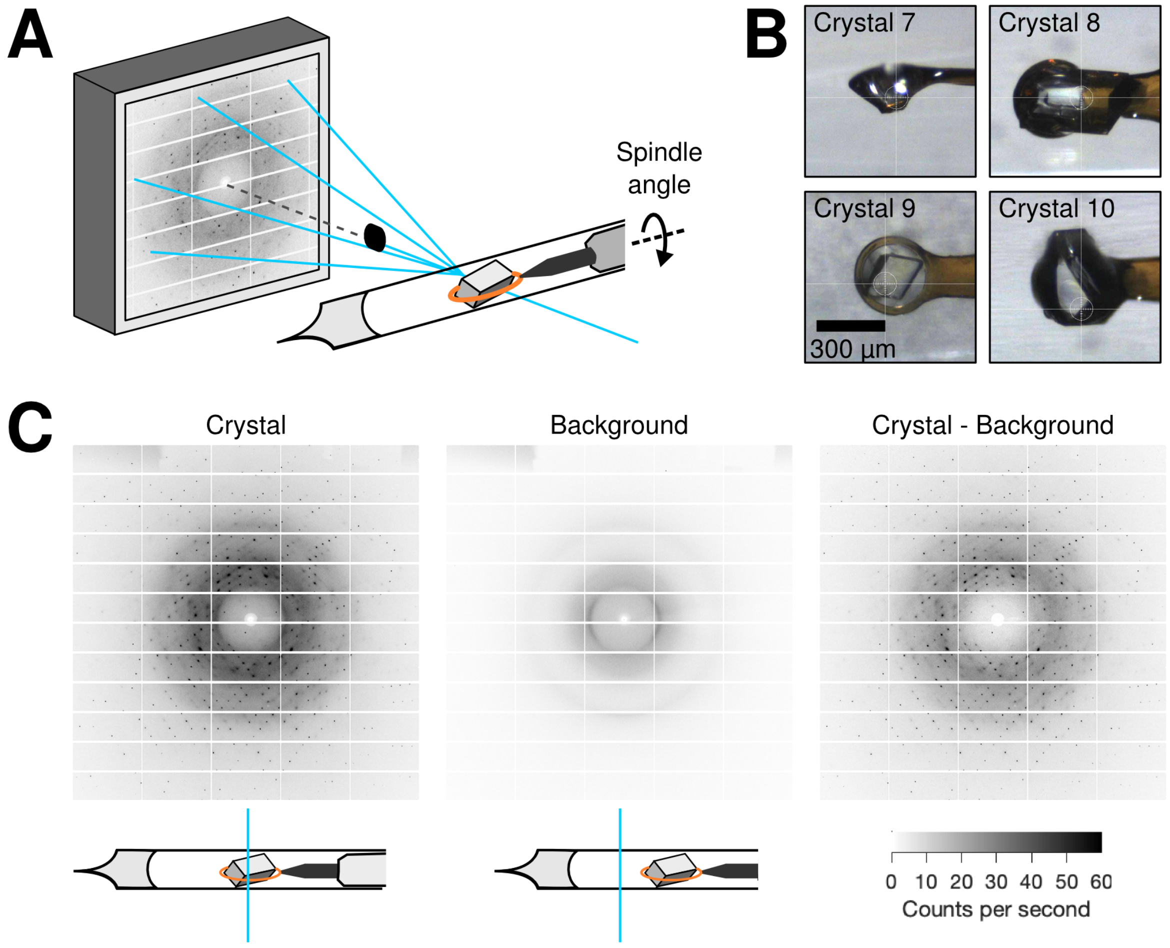

Supplementary Fig. 2: X-ray data collection strategy. (A) Diagram of the experimental setup. To prevent dehydration at ambient temperature, each crystal was enclosed in a plastic capillary with crystallization solution in the tip. X-ray diffraction images were recorded by a photon-counting detector using shutterless data collection with fine phi-slicing (0.1° per frame). (B) The total dose was distributed over 11 diffraction volumes from four large crystals (Supplementary Table 1). (C) At each spindle angle (phi), diffraction images were collected with the crystal in the beam (left) and with the crystal translated out of the beam (middle). As the background includes scattering from the instrument and narrow rings due to the plastic capillary, the pattern due to the sample only is estimated by subtraction (right). For clear illustration, the images shown are the sums of 10 sequential frames (1° oscillation and 1 s exposure in total).

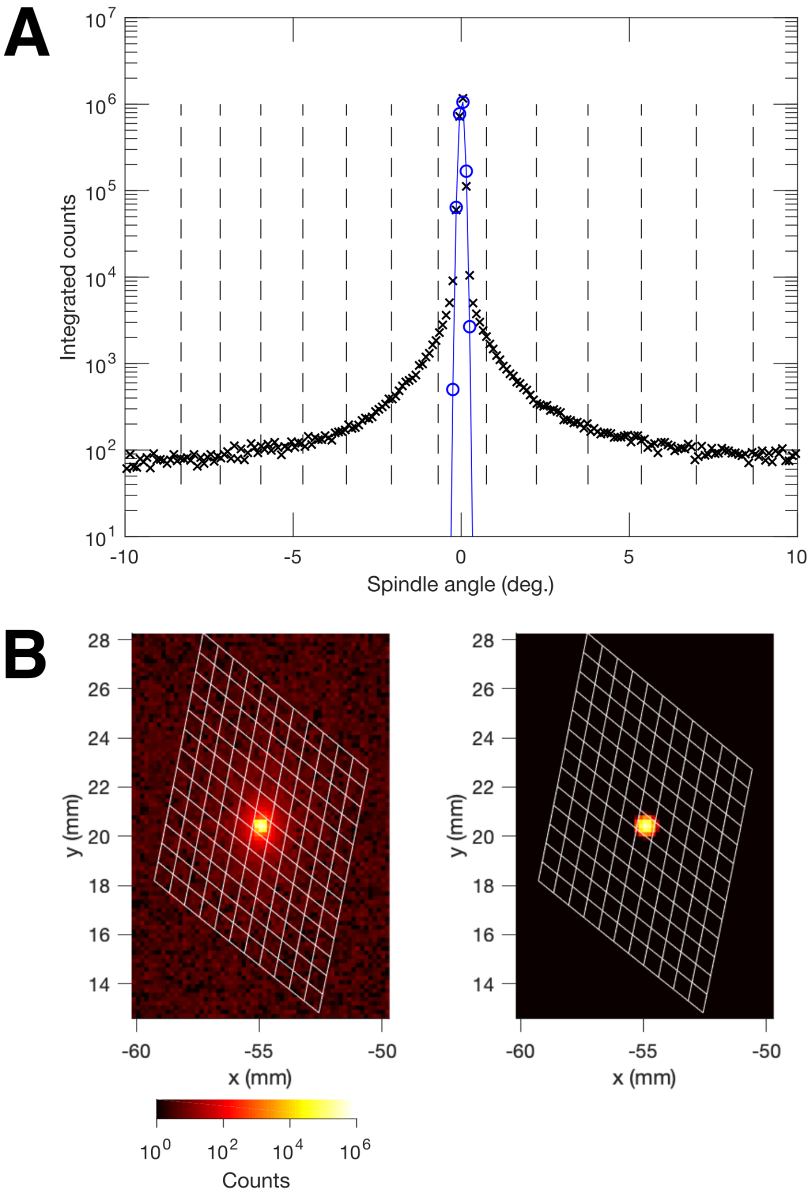

Supplementary Fig. 3: Rocking curve for the (2,-8,-1) reflection. In our diffraction data from triclinic lysozyme, the apparent crystal mosaicity of 0.02 to 0.03° is smaller than the oscillation width of 0.1° per frame, so most Bragg reflections appear only in one or two consecutive images and their rocking curves cannot be resolved. We thus identified an intense (2,-8,-1) Bragg reflection that is close to the spindle axis and passes through the Ewald sphere along $\mathbf{c}^*$ at a rate approximately 3 times slower than the fastest route. The peak profile above the background was well-fit by a three-dimensional Gaussian model [2] with apparent mosaicity and beam divergence of 0.020° and 0.024°, respectively. (A) The total photon counts in a 25-pixel region around the Bragg peak is plotted vs. spindle angle from the diffraction maximum (black symbols). The sharp Bragg peak (Gaussian fit shown in blue) is easily distinguished from the more slowly-varying background. The vertical dashed lines show the voxel subdivisions along $\mathbf{c}^*$ used in the construction of the fine diffuse map. The peak is confined to the central subdivision. (B) On the left is a zoom-in of the (2,-8,-1) Bragg reflection in the diffraction image (0.1° oscillation) at its peak intensity. The x and y axes are distances from the beam center, and the spindle is parallel to the x-axis. On the right is the same oscillation simulated using the Gaussian model. In both images, the intersection of voxel edges in the $\mathbf{a}^*$-$\mathbf{b}^*$ plane with the Ewald sphere are projected onto the detector plane, showing that the Bragg intensity is confined to the central voxel.

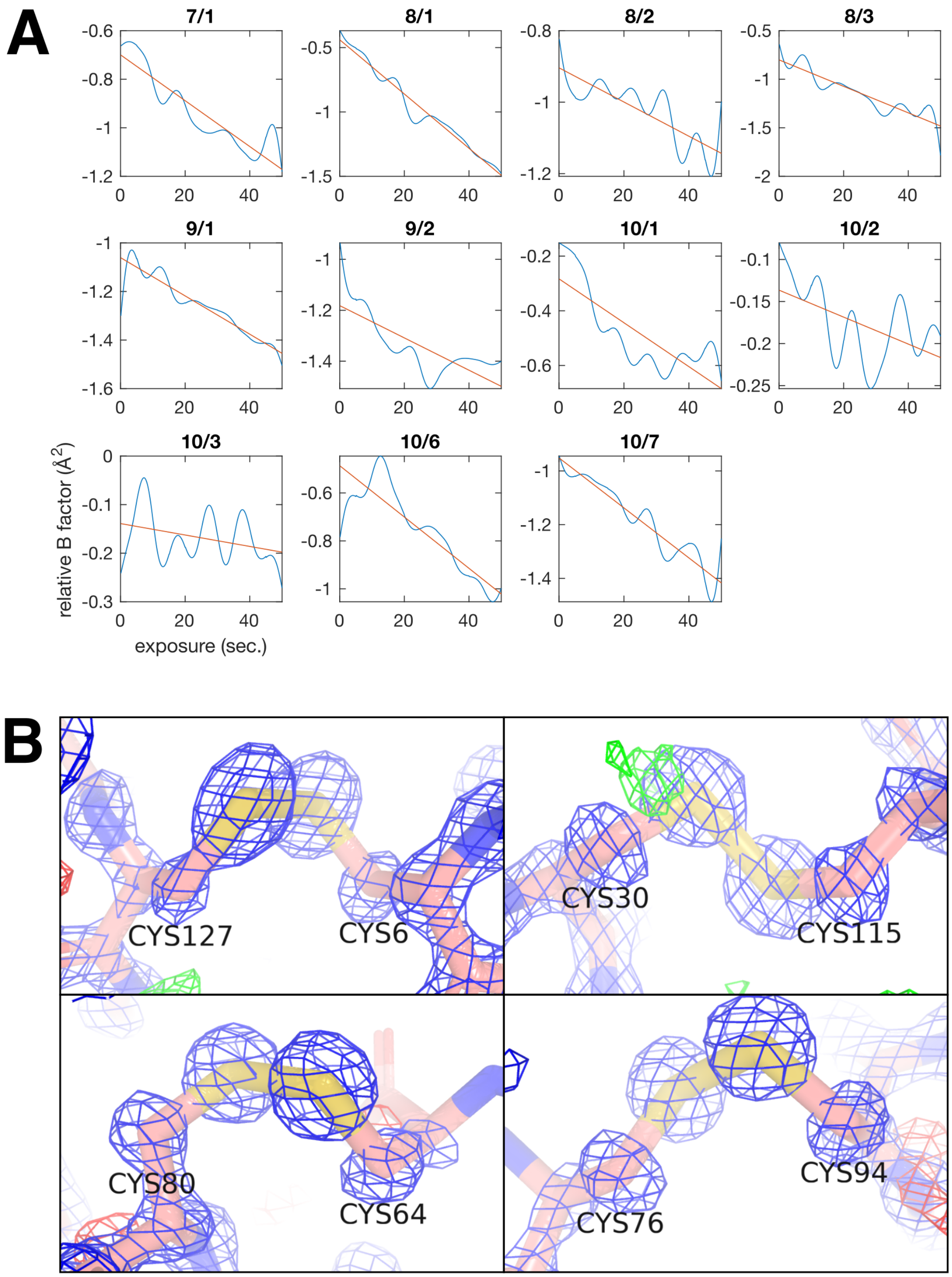

Supplementary Fig. 4: Minimal radiation damage is observed in the triclinic lysozyme dataset. (A) In general, global radiation-induced damage manifests as a decay in the Wilson B-factor with increasing dose, which is corrected by a relative B-factor when scaling Bragg data with *aimless* [3]. The relative B-factor (blue curves) and a linear fit (red lines) are shown for all 11 diffraction volumes from four crystals (Supplementary Fig. 2B) with labels corresponding to crystal number / wedge number (Supplementary Table 1). For each wedge, the Wilson B-factor decays by less than 1 $Å^2$ over the course of the measurement. (B) Disulfide bonds were also inspected for evidence of photo-reduction in the structure determined from the combined data. The four disulfide bonds in the lysozyme are shown along with the 2Fo-Fc map (blue mesh at 2.5σ) and the Fo-Fc map (red and green mesh at $\pm 2.5\sigma$). The bonds appear largely intact. The difference density near the sulfur atom of C30 (top right panel) may indicate that a minor population of C30-C115 has been reduced.

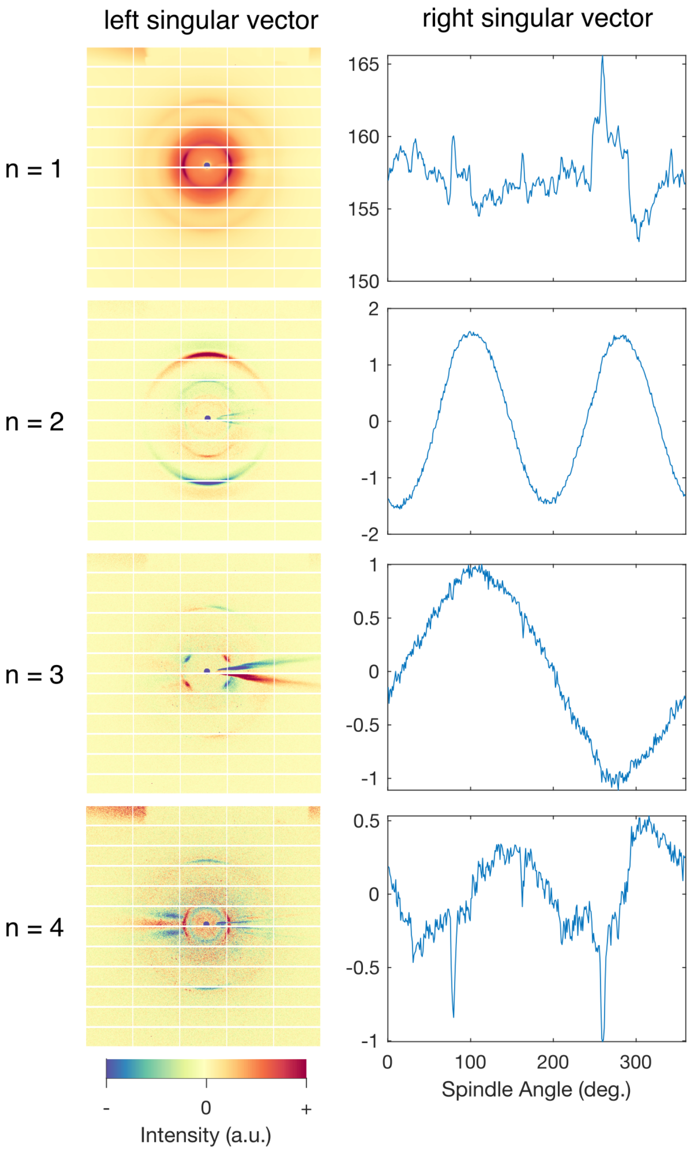

Supplementary Fig. 5: Importance of background subtraction. Singular value decomposition of the set of background images (360°, 1 s exposures, 1° oscillation) shows several scattering features that vary independently as the spindle rotates. The first four singular vectors are shown (n = 1 to 4); the left singular vectors correspond to diffraction images, and the right singular vectors show the contribution as a function of spindle angle. The $n = 1$ component contains the overall background scattering. The $n = 2$ component, which has ring-like features and a period of 180°, can be attributed to anisotropic scattering of the capillary. The $n = 3$ component, with strong equatorial features and a period of 360°, is due to the shadow cast by the sample pin as it precesses. The remaining components ($n > 3$) have features due to both the pin shadow and the capillary, as well as experimental noise.

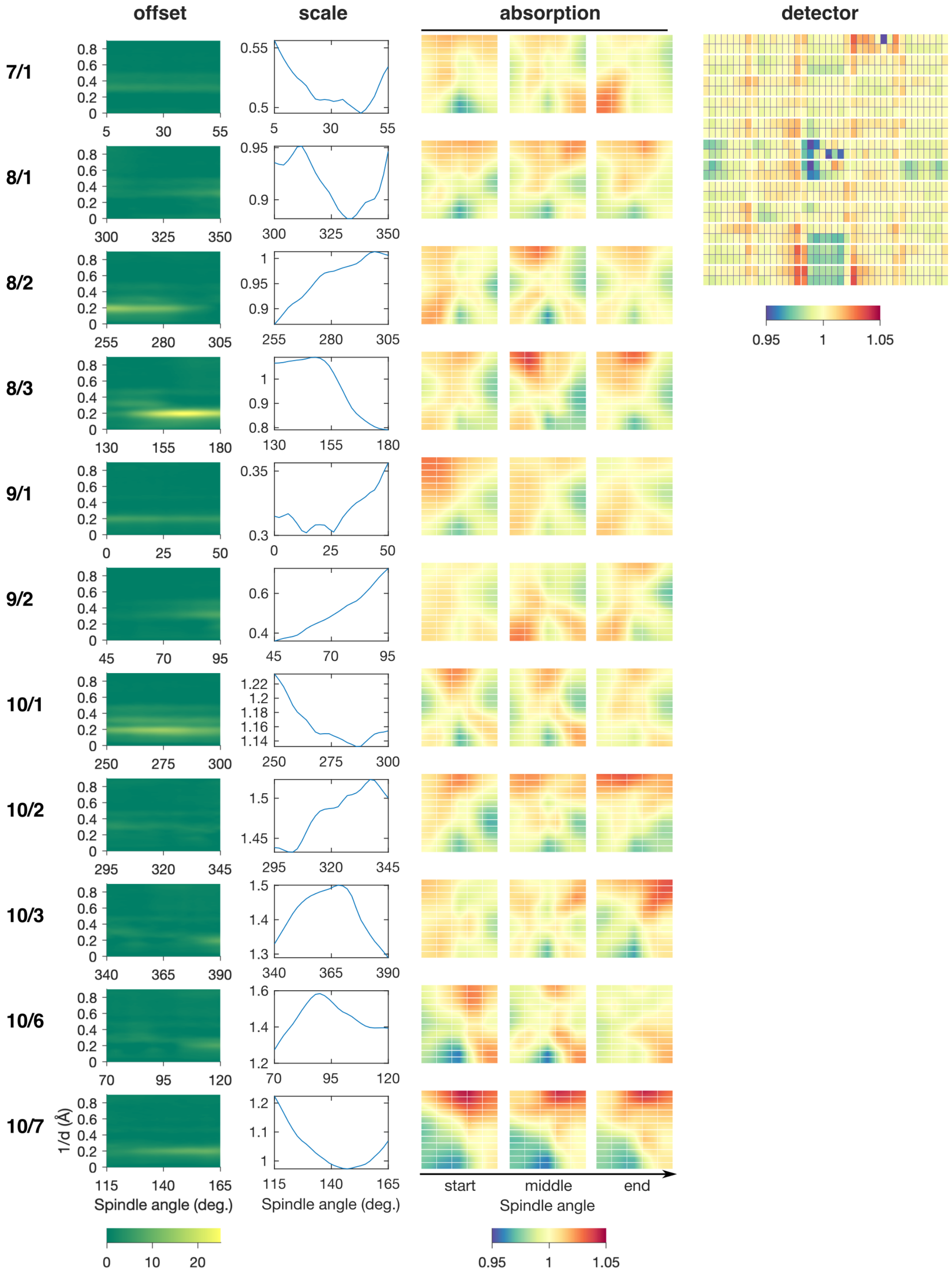

Supplementary Fig. 6: Refinement of a scaling model to correct for experimental artifacts in X-ray images. The scaling model (Equation 1 in the Main Text) used in processing diffuse scattering data relates the merged intensity to the observed intensity as a function of spindle angle, resolution (d), and detector position. From left to right, the model parameters included: (1) offset correction vs. spindle angle and resolution, (2) overall scale factor vs. spindle angle, (3) scale factor for absorption correction vs. spindle angle and detector position, and (4) scale factor for detector efficiency correction vs. detector chip index. The parameters in the model were refined in order to minimize the least-squares error between observation and prediction (Equation 2 in the Main Text). The best-fit-parameters are illustrated for each partial dataset (top to bottom) labeled according to crystal number / wedge number (refer to Supplementary Fig. 2B, Supplementary Table 1).

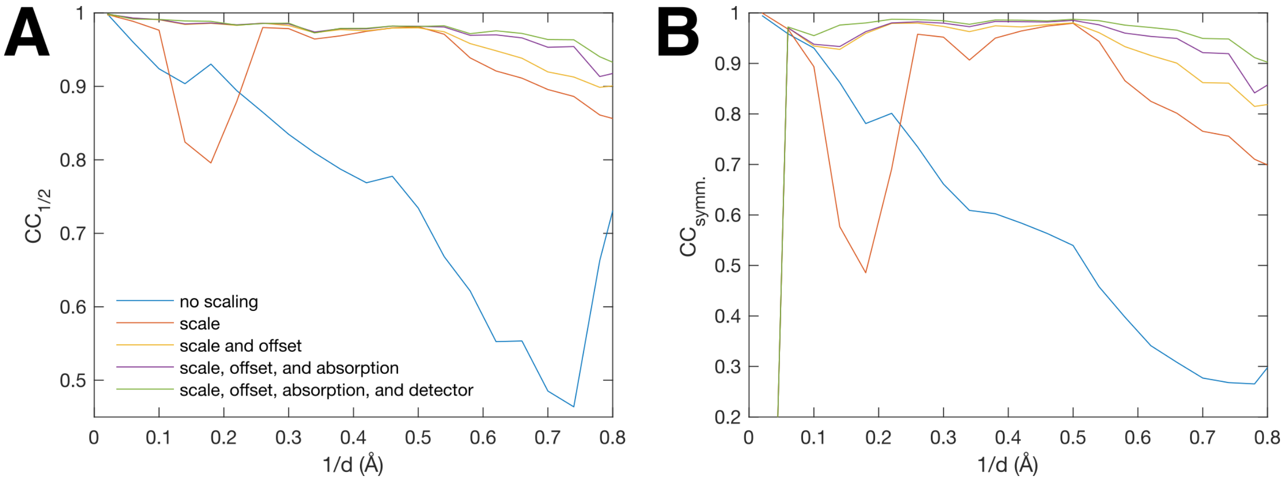

Supplementary Fig. 7: Impact of scaling parameters on diffuse data quality. Four parameters were refined sequentially to the coarse continuous scattering map (one point per integer h,k,l): scale, offset, absorption, and detector (labeled in Supplementary Fig. 6 and described in the Methods section of the Main Text). Data quality was assessed by comparing Pearson correlation coefficients (CC) between half-datasets within shells of constant resolution. (A) CC between random half-datasets ($CC_{1/2}$). (B) CC between half-datasets related by Friedel symmetry ($l > 0$ and $l < 0$, $CC_{symm.}$). Both metrics show improvements in data quality as each parameter is added.

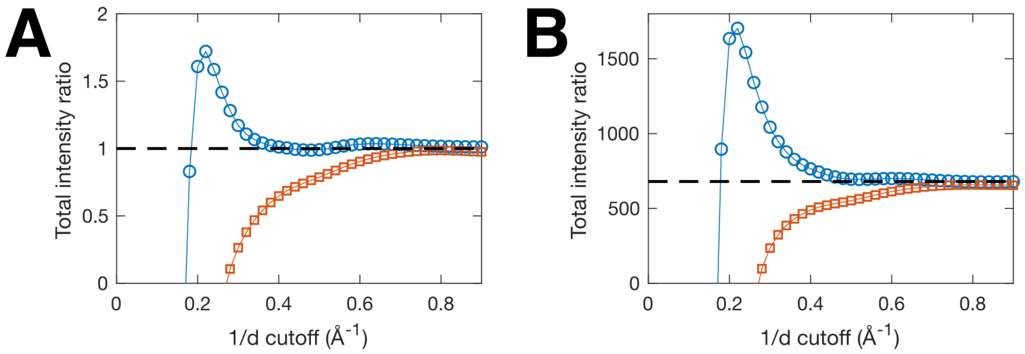

Supplementary Fig. 8: Robustness of absolute intensity calibrations performed with the Krogh-Moe (K-M) method (red squares) and the modified K-M method, which accounts for an interference term that depends on bond geometries (blue circles). (A) A test dataset of X-ray intensities (Bragg, diffuse, and Compton) was calculated from the 1-unit cell MD simulation of triclinic lysozyme, and the ratio between predicted and actual total intensity was plotted vs. resolution cutoff using each method. Both converge to the expected value of 1 (dashed line), but the modified method converges much more quickly. (B) The same methods applied to the experimental maps of continuous scattering and Bragg diffraction on an arbitrary intensity scale. The modified K-M method converged by ~ 2 Å resolution, yielding a scale factor of 680 (dashed line).

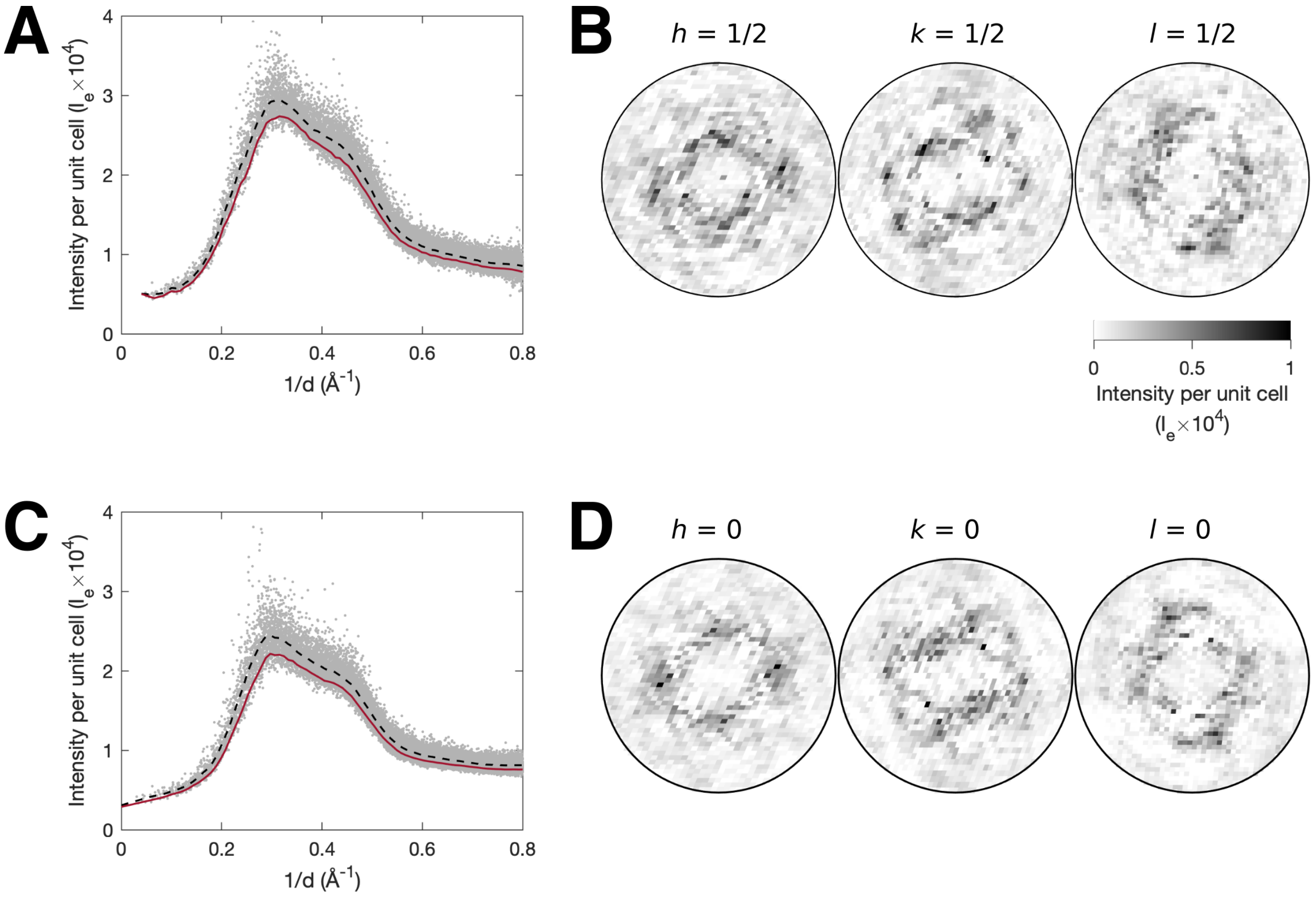

Supplementary Fig. 9: Isotropic component of diffuse scattering in experiment and the 1-unit cell MD simulation. (A) The diffuse scattering was resampled at half-integer values of h, k, and l, where halo scattering is minimal. To improve the signal-to-noise ratio, the interpolation was performed by least-squares fitting a 2nd order polynomial to the 5x5x5 voxel region around each point in the target grid. The resulting "half-integer map" (gray points) was analyzed as a function of the resolution d. Within each resolution bin, the isotropic background (red curve) was defined as 1σ below the mean (dashed black curve). (B) The isotropic background was subtracted from the total intensity to aid in visualizing the cloudy variational pattern. Slices from left to right are perpendicular to $\mathbf{a}^*$, $\mathbf{b}^*$, and $\mathbf{c}^*$. (C-D) The 1-unit cell MD simulation was analyzed in the same way as the half-integer map. The isotropic scattering and variational scattering components are similar between the 1-unit cell MD simulation and the half-integer map.

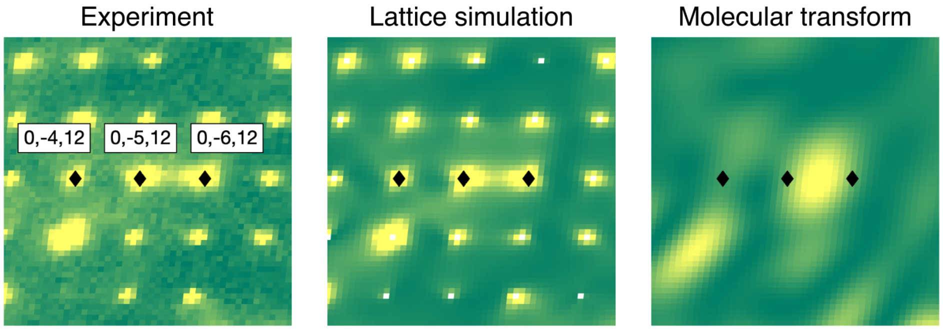

Supplementary Fig. 10: Diffuse streaks attributed to intense features in the molecular transform. In the experimental maps of variational scattering (left panel), certain pairs of halos are connected by streaks of diffuse intensity, such as (0,-5,12) and (0,-6,12), while others are not, such as (0,-4,12) and (0,-5,12). Similar features are seen in the one-phonon scattering simulation of the lattice dynamics model (center panel). These anisotropic features occur because the halo intensities are modulated by the molecular transform, the continuous diffraction intensity of an isolated unit cell (right); the molecular transform is intense between (0,-5,12) and (0,-6,12) but is close to zero between (0,-4,12) and (0,-5,12), explaining the presence of a diffuse streak in the former case and its absence in the latter.

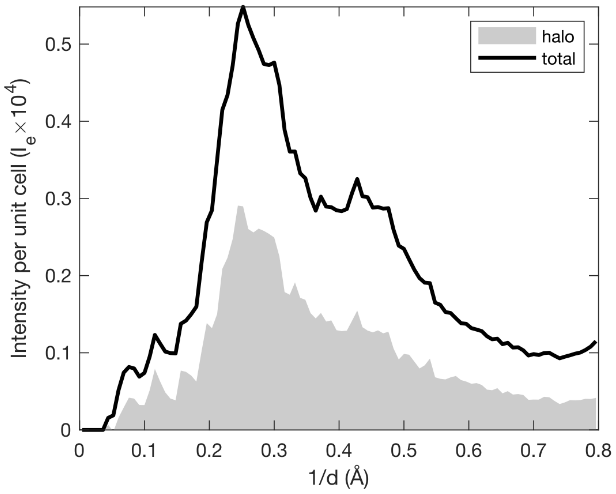

Supplementary Fig. 11: Contribution of halos to the total variational scattering. The mean variational intensity in each resolution shell was calculated for the full map, which includes the halo features, and for the half-integer interpolated map (described in Supplementary Fig. 9A), which does not. The halo contribution was estimated as the difference between the two (shaded region). In most resolution shells, the halos account for about half of the total variational intensity (solid curve).

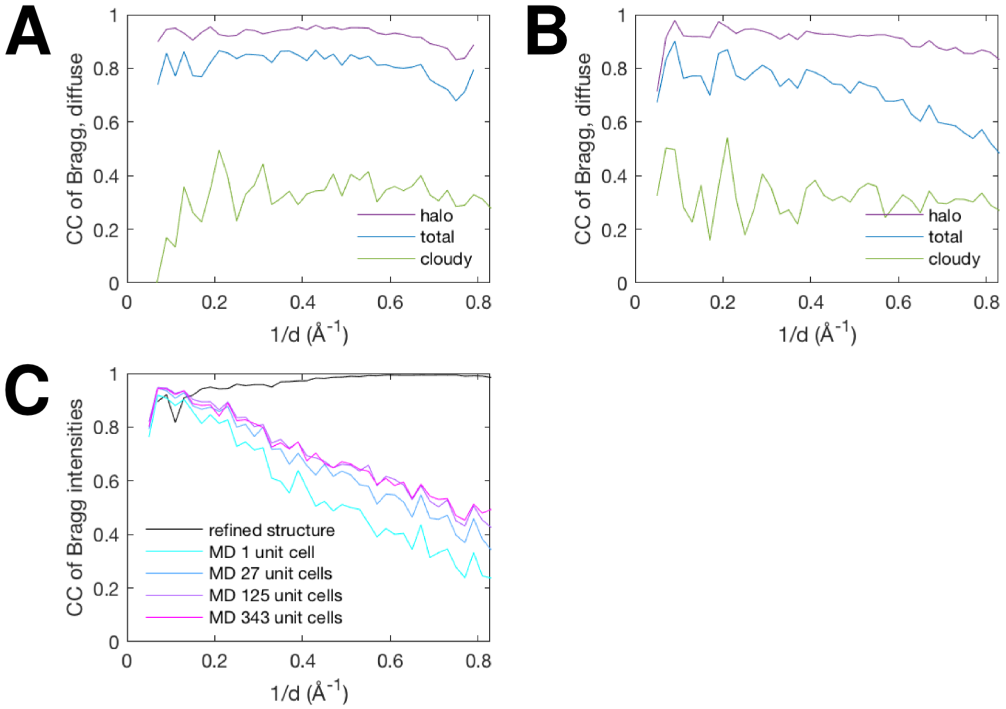

Supplementary Fig. 12: Correlations between Bragg and diffuse intensities. The total variational intensity, which includes halos and cloudy scattering, was averaged in neighborhoods (h,k,l) around each Bragg peak (h_0,k_0,l_0), where $|h-h_0| < 1/2$, $|k-k_0| < 1/2$, and $|l-l_0| < 1/2$. The contribution of cloudy scattering was estimated by linear interpolation of the half-integer map (Supplementary Fig. 9A). Halo scattering was estimated by subtracting the cloudy scattering from the total variational intensity. (A) The Pearson correlation coefficients (CC) were calculated between the Bragg intensities and different components of diffuse scattering: total variational scattering (blue curve), cloudy scattering (green curve), and halo scattering (purple curve). The correlations are positive in all resolution shells, with halo intensities showing the strongest correlation. (B) The diffuse scattering and Bragg intensities calculated from the 343-unit cell MD simulation were analyzed as in panel A. The Bragg intensities and halo intensities are also strongly correlated. (C) CC of Bragg intensities between experiment and four MD simulations with 1-343 unit cells (colored curves, see legend). The CC is strong at low resolution, but decays significantly at high resolution. In contrast, the CC between the experiment and the refined crystal structure model (black curve) remains excellent at high resolution.

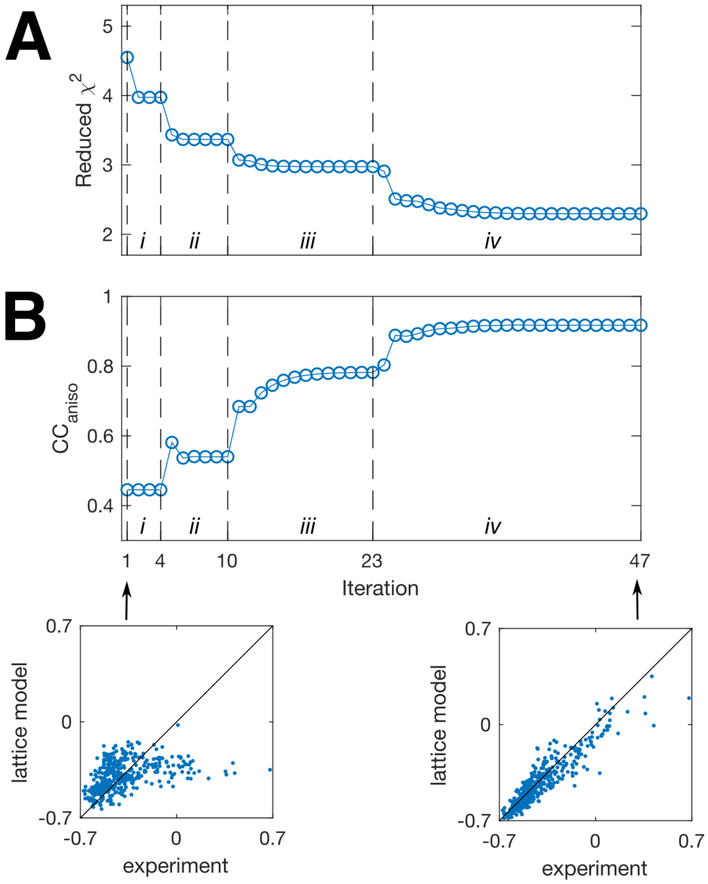

Supplementary Fig. 13: Refinement of lattice dynamics model against a small subset of diffuse halos. (A) The spring constants in the lattice dynamics model were refined iteratively to minimize the least-squares difference (reduced χ^2, blue symbols) between the calculated one-phonon intensity and the measured variational intensity around 400 intense halos. To avoid over-fitting, the refinement was carried out in four stages (i-iv) as restraints were removed (see Methods in the Main Text). The first stage (i) is a Gaussian model with equal springs, while in the final stage (iv) all springs are refined individually. (B) The ability of the model to explain halo anisotropy (see Fig. 3C in the Main Text) was assessed using the Pearson correlation coefficient for the 400 halos (CC_{aniso}). Initially, the anisotropy parameters for model and experiment show little correlation ($CC_{aniso} = 0.45$, scatter plot in lower left). After refinement, the correlation improves ($CC_{aniso} = 0.92$, scatter plot in lower right).

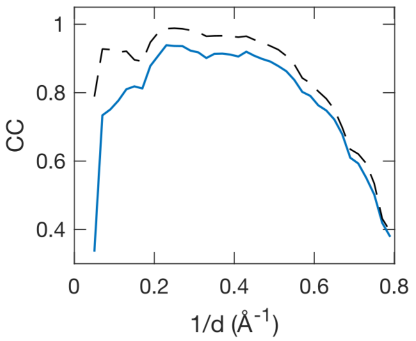

Supplementary Fig. 14: Correlation of experiment and simulated one-phonon scattering from the refined lattice dynamics model calculated with intensities from the full 13x11x11 grid. The data-model correlation (solid blue curve) is excellent (CC $\sim$ 0.9) between 2 and 5 Å resolution (solid), but decays rapidly beyond $\sim$ 2 Å resolution. At high resolution, the correlation is limited by noise in the experimental map, as estimated using the CC* statistic (dashed curve). Interpolating the maps on a 7x7x7 grid improves the signal-to-noise (see Fig. 2D in the Main Text).

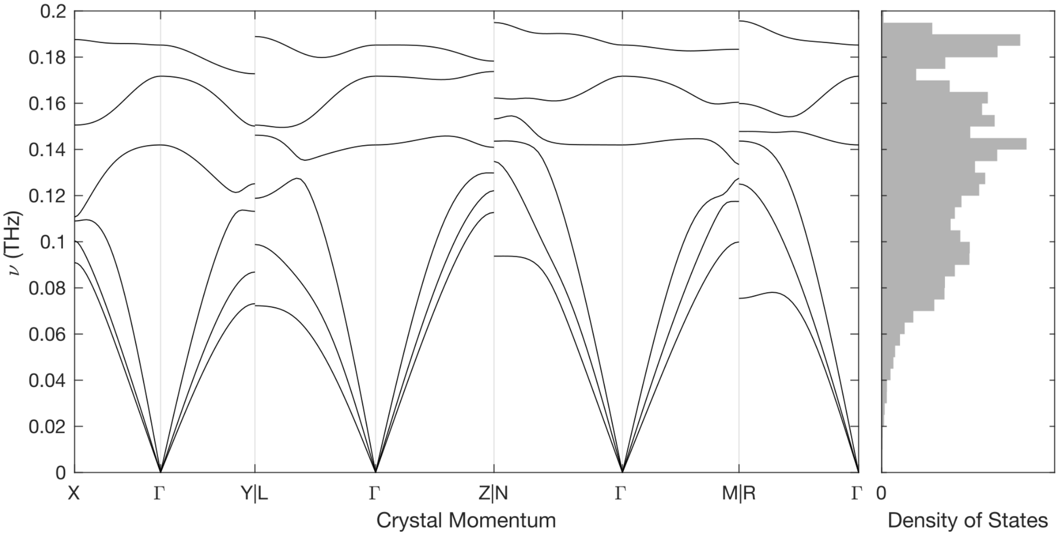

	b_1	b_2	b_3
Γ	0	0	0
L	0	0.5	-0.5
M	0.5	0	0
N	0.5	-0.5	-0.5
R	0.5	0	-0.5
X	0	0	-0.5
Y	0	0.5	0
Z	0.5	-0.5	0

Supplementary Fig. 15: Dispersion relations for lattice modes of triclinic lysozyme. The vibrational frequencies ($\nu = 2\pi\omega$) were calculated from the refined lattice dynamics model along a path through k-space (x-axis on the left plot) that passes through the origin (Γ) as well as special points at the edge of the Brillouin zone (defined in the table on the right, where b_1, b_2, and b_3 are the fractional coordinates along a*, b*, and c*). The vibrational density of states (right plot) was calculated for 11,025 evenly spaced points in the Brillouin zone (a 25x21x21 supercell). In the model, each protein chain has 6 degrees of freedom (3 coordinates each for translations and rotations), resulting in 6 vibrational modes: 3 optical and 3 acoustic. The acoustic modes are those that pass through the origin.

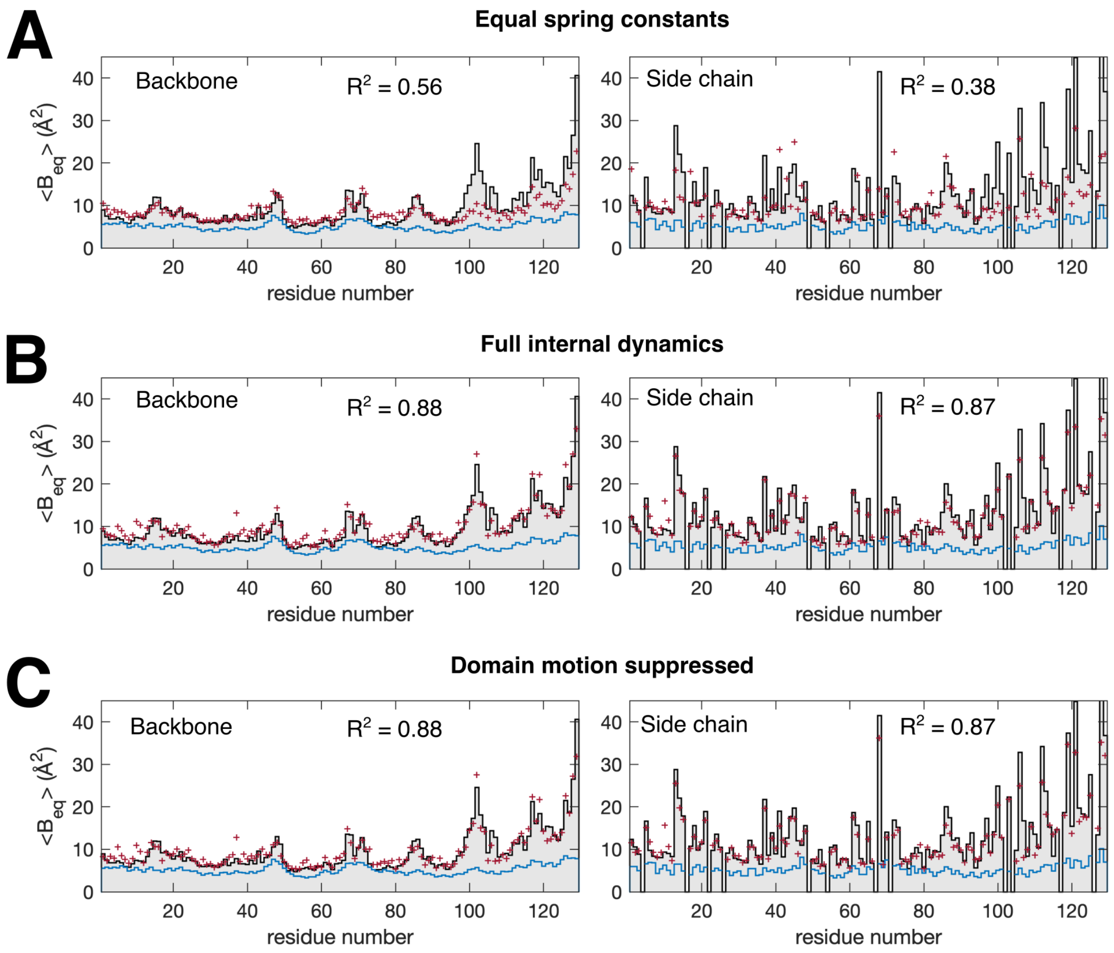

Supplementary Fig. 16: Elastic network models of internal motion refined to the atomic displacement parameters (ADPs). For visual clarity, the average equivalent isotropic B-factors were computed for non-H atoms in the backbone and side-chains for the refined crystal structure (black curve and gray shaded area), the lattice dynamics model (blue curve), and the lattice + internal dynamics models (dark red symbols). (A) A model with equal spring constants reproduces many of the backbone features (left), except at the C-terminus (residues 95-129). Many of the side-chain B-factors are not reproduced (right). (B) The B-factors are reproduced by refining the spring constants in a model parameterized with one coupling constant per residue (see Fig. 4C in the Main Text). Agreement for the backbone and side-chain B-factors is improved. (C) Repeating the refinement in panel B with a modified potential energy function, which suppresses collective motion of the α and β domains (see Fig. 4D and Methods in the Main Text), lead to a similarly reasonable fit to the B-factors.

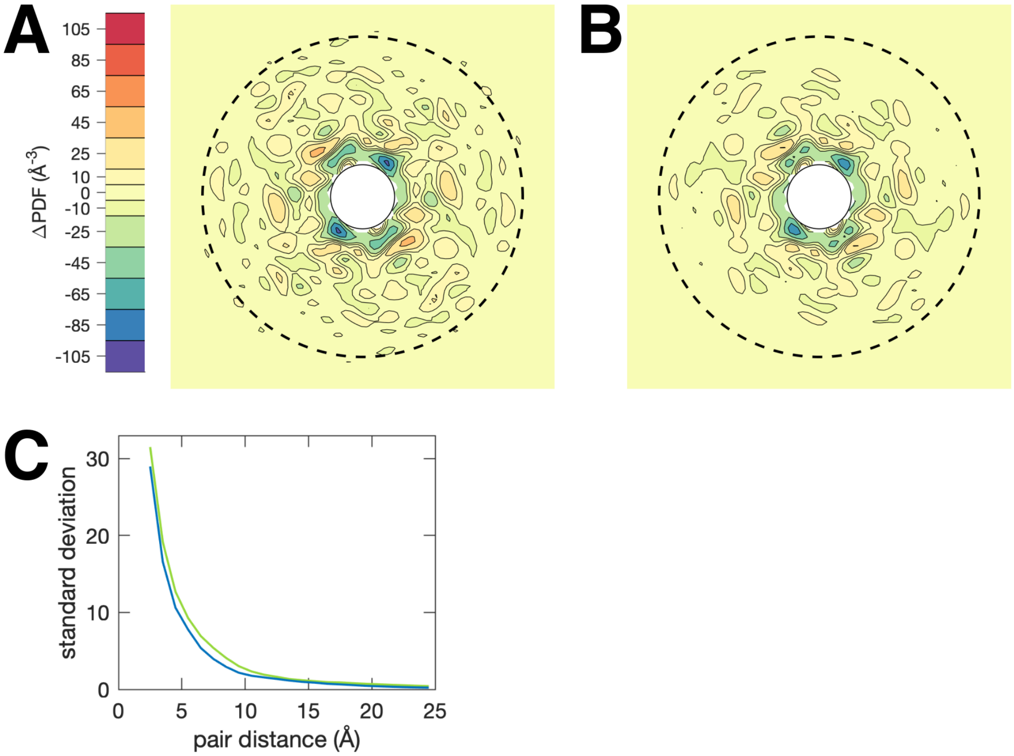

Supplementary Fig. 17: Comparison of models for internal motion by diffuse Patterson analysis. The central sections of the diffuse Patterson maps in the in **a**-**b** plane (dashed circle corresponds to 10 Å) are shown for the two elastic network models that fit equivalently well to the experimental ADPs (Supplementary Fig. 16B-C). (A) Fluctuations are observed to $\sim$ 10 Å when full internal motions are allowed (figure panel repeated from Fig. 5C in the Main Text). (B) Suppressing collective motion of the α and β domains in lysozyme leads to a diffuse Patterson map that is significantly altered. (C) The amplitudes of the fluctuations in the diffuse Patterson for the no-domain-motion model (blue curve) decay more rapidly with pair distance than those of the unconstrained model (green curve, repeated from Fig. 5F in the Main Text).

Supplementary Tables

Crystal	Wedge	Frames	Spindle Range (deg.)	$\langle I/\sigma I \rangle$
7	1	1-500	5-55	16.0
8	1	1-500	300-350	14.0
	2	1-500	255-305	15.6
	3	1-500	130-180	14.6
9	1	1-500	0-50	18.7
	2	1-500	45-95	18.1
10	1	1-500	250-300	16.2
	2	1-500	295-345	23.1
	3	1-500	340-390	18.7
	6	1-500	70-120	20.3
	7	1-500	115-165	14.9

Supplementary Table 1: The X-ray datasets that were combined and used for both structure determination and diffuse scattering analysis in this study.

	PDB ID *6o2h*
**Data collection**	
Space group	P 1
a, b, c (Å)	27.42, 32.13, 34.51
α, β, γ (°)	88.66, 108.46, 111.88
Mosaicity (°)	0.030
Resolution range (Å)	32.55–1.21 (1.23-1.21)[a]
R_{pim}	0.025 (0.038)
$\langle I/\sigma(I)\rangle$	27.4 (12.5)
$\mathrm{CC}_{1/2}$	0.995 (0.994)
Completeness (%)	96.1 (52.9)
Multiplicity	4.7 (1.4)
**Refinement**	
Resolution (Å)	32.55–1.21
Unique reflections: all / free	30108 / 1495
R_{work}/ R_{free}	0.096 / 0.117
Number of non-H atoms	
Protein	1122
Ligand/Ion	29
Water	92
Mean isotropic B-factors	
Protein	12.45
Ligand/Ion	31.44
Water	26.25
Model validation[b]	
Ramachandran outliers (%)	0
Ramachandran favored (%)	99.21
Rotamer outliers (%)	0.81
C-β deviations	0
R.m.s. bond lengths (Å)	0.0272
R.m.s. angles (°)	2.31
Clashscore	0.00
Overall score	0.50

[a]Values in parentheses are for highest-resolution shell.
[b]Calculated using MolProbity[4]

Supplementary Table 2: Data collection and refinement statistics.

Wavevector direction	Longitudinal fraction (%)	v_s (m/s)
Γ-L	7.2	601.2
	12.1	819.6
	99.1	1270.3
Γ-M	1.0	744.4
	18.1	606.1
	98.3	1033.4
Γ-N	20.6	675.7
	28.0	876.5
	93.6	1170.9
Γ-R	18.4	852.6
	22.8	769.1
	95.7	1202.6
Γ-X	1.7	694.0
	22.7	775.9
	97.2	1294.0
Γ-Y	26.9	708.5
	51.3	598.1
	81.8	1059.2
Γ-Z	0.9	693.5
	9.4	793.0
	99.6	1047.4

Supplementary Table 3: Acoustic phonon properties. Wavevector directions are defined in Supplementary Fig. 15. Longitudinal fraction and speed of sound (v_s) were calculated for each of the three acoustic phonons at a fractional wavevector magnitude of 0.1. The longitudinal fraction [5] was defined as $(\hat{\mathbf{u}} \cdot \hat{\mathbf{k}}) \times 100\%$, where $\mathbf{k}$ is the wavevector and $\mathbf{u}$ is the principal axis of vibration of the protein center of mass.

Protein center of mass (x,y,z) (Å)	(-0.943, 14.007, 24.322)
Center of reaction (x,y,z) (Å)	(-1.844, 13.061, 25.358)
$\mathbf{T}$ ($T_{1,1}$, $T_{2,2}$, $T_{3,3}$, $T_{1,2}$, $T_{1,3}$, $T_{2,3}$) (Å^2)	(0.0385, 0.0443, 0.0377, 0.0009, 0.0037, 0.0024)
$\mathbf{L}$ ($L_{1,1}$, $L_{2,2}$, $L_{3,3}$, $L_{1,2}$, $L_{1,3}$, $L_{2,3}$) (deg.^{2})	(0.5675, 0.7941, 0.6688, -0.0789, -0.0383, -0.1438)
$\mathbf{S}$ ($S_{1,1}$, $S_{2,2}$, $S_{3,3}$, $S_{1,2}$, $S_{1,3}$, $S_{2,3}$) (deg.Å)	(0.0109, -0.0075, 0.0034, -0.0103, -0.0012, -0.0079)
r.m.s. displacement = $(\mathrm{tr}(\mathbf{T})/3)^{1/2}$ (Å)	0.200
r.m.s. rotation = $(\mathrm{tr}(\mathbf{L})/3)^{1/2}$ (deg.)	0.823

Supplementary Table 4: Translation libration screw (TLS) parameters [6] calculated from the refined lattice dynamics model (Equation 58 in the Supplementary Methods). The center of reaction was chosen as the origin for the rigid group, which makes **S** symmetric and minimizes the trace of **T**.

Supplementary Notes

Supplementary Note 1. Number of free parameters in the elastic network model

Two elastic network models for internal protein motion were fit to the residual ADPs. The number of normal modes for the elastic network is $129 \times 6 - 6 = 771$, since there are 129 rigid groups (one per residue) with 6 degrees of freedom each, and the restraint that there is no rigid-body motion of the entire protein reduces the number of modes by 6. In the model with domain motion suppressed, similar restraints applied to the 3 rigid groups (α, β, and hinge) result in $129 \times 6 - 3 \times 6 = 756$ normal modes. Thus, by restraining 3 groups instead of 1, the number of normal modes is very moderately reduced, which in principle might make it more difficult to fit the ADPs. However, we did not adjust the normal mode amplitudes to fit the ADPs as was done in prior studies [7, 8]. Instead, we refined the physical model (spring constants) through a conservative parameterization with one coupling constant per residue (or 129 free parameters, see Equation 12 in the Main Text). Thus, the ability of each model to fit the ADPs is not limited by the number of normal modes, but is instead limited primarily by the parameterization, which is the same in both cases.

Supplementary Methods

1 General Theory

The kinematical theory of X-ray diffraction applicable to diffuse scattering from proteins has been described previously [9, 10]. Here we provide an overview of the main results as relevant to this study.

1.1 Total scattering cross-section

The macroscopic differential scattering cross-section, $d\Sigma/d\Omega$, describes the probability of observing a scattering event in the solid angle $d\Omega$ when the sample is illuminated by X-rays. The cross-section depends on the respective wavevectors of the incident and scattered X-rays, $\mathbf{s}_0$ and $\mathbf{s}'$. The wavevectors have magnitudes of inverse wavelength: $s_0 = |\mathbf{s}_0| = 1/\lambda$ and $s' = |\mathbf{s}'| = 1/\lambda'$, where λ and λ' are the wavelengths of the incident and scattered X-ray photons. Given an incident flux, J_0, the total scattered flux into a solid angle $\Delta\Omega$ in the direction $\hat{s}'$ is as follows:

$$J(\hat{\mathbf{s}}') = J_0 \Delta\Omega \frac{d\Sigma}{d\Omega}(\mathbf{s}_0, \mathbf{s}'). \tag{1}$$

In the X-ray experiments described here, both coherent and incoherent processes contribute to X-ray scattering:

$$\frac{d\Sigma}{d\Omega} = \left(\frac{d\Sigma}{d\Omega}\right)_{\text{coherent}} + \left(\frac{d\Sigma}{d\Omega}\right)_{\text{incoherent}}. \tag{2}$$

The coherent X-ray scattering has a cross-section

$$\left(\frac{d\Sigma}{d\Omega}\right)_{\text{coherent}} = I_e(\hat{\mathbf{s}}_0, \hat{\mathbf{s}}') I_{\text{sample}}(\mathbf{s}), \tag{3}$$

where $\mathbf{s} = \mathbf{R}^{\mathrm{T}}(\mathbf{s}' - \mathbf{s}_0)$ is the scattering vector in the sample frame which is rotated with respect to the lab frame by a rotation matrix $\mathbf{R}$, I_{sample} is the coherent intensity of the sample in per-electron units, and I_e is the Thomson scattering cross-section of a free electron,

$$I_e = r_e^2 P(\hat{\mathbf{s}}_0, \hat{\mathbf{s}}'), \tag{4}$$

where r_e is the classical electron radius and $P(\hat{\mathbf{s}}_0, \hat{\mathbf{s}}')$ is the polarization factor.

Incoherent, or Compton, X-ray scattering has a cross-section [11]:

$$\left(\frac{d\Sigma}{d\Omega}\right)_{\text{incoherent}} = I_e(\hat{\mathbf{s}}_0, \hat{\mathbf{s}}') S_{\text{sample}}(\mathbf{s}), \tag{5}$$

where S_{sample} is the sum of atomic incoherent scattering functions over all illuminated atoms[12]. The Compton scattering process is inelastic: the scattered photon has less energy than the incident photon by an amount that increases with scattering angle. However, the wavelength shift, which is at most twice the Compton wavelength of the electron $\lambda_C = h m_e^{-1} c^{-1} \approx 0.02426\,\text{Å}$, is small compared with the $\lambda \sim 1\,\text{Å}$ wavelengths generally employed in X-ray crystallography. Therefore, in the following, we assume $\lambda = \lambda'$.

The sample in this case is a crystal composed of unit cells. The crystal is assumed to be homogeneous, meaning that the unit cells are equivalent to each other on average, and therefore one can write:

$$I_{\text{sample}}(\mathbf{s}) = (V_0/v_c) I(\mathbf{s}) \tag{6}$$

and

$$S_{\text{sample}}(\mathbf{s}) = (V_0/v_c) S(\mathbf{s}), \tag{7}$$

where V_0 is the volume of the sample illuminated by the X-ray beam, v_c is the unit cell volume (V_0/v_c is the number of unit cells illuminated), and $I(\mathbf{s})$ and $S(\mathbf{s})$ are the coherent and incoherent intensities *per unit cell*. Finally, we can write the scattered flux in terms of the intensities per unit cell by combining Equations 1-7, as follows:

$$J(\hat{\mathbf{s}}') = J_0 \Delta\Omega r_e^2 P(\hat{\mathbf{s}}_0, \hat{\mathbf{s}}') (V_0/v_c) \left[I(\mathbf{s}) + S(\mathbf{s})\right]. \tag{8}$$

1.2 Coherent scattering from crystals

Coherent scattering of X-rays depends on the spatial distribution of electrons in the sample. If $\rho(\mathbf{r})$ is the instantaneous number density of electrons at a position $\mathbf{r}$, the scattering is described by the Fourier transform of the electron density, a quantity known as the structure factor, $F(\mathbf{s})$:

$$F(\mathbf{s}) = \int \rho(\mathbf{r}) e^{2\pi i \mathbf{s}\cdot\mathbf{r}} d^3\mathbf{r}. \tag{9}$$

The intensity per unit cell is proportional to the mean square structure factor, as follows:

$$I(\mathbf{s}) = N^{-1} \left\langle F(\mathbf{s})^2 \right\rangle, \tag{10}$$

where N is the number of unit cells under consideration and brackets denote the ensemble (or time) average. In general, the electron density is time-dependent, but the system is in equilibrium so that we can define an average electron density $\langle \rho(\mathbf{r}) \rangle$ with corresponding structure factor $\langle F(\mathbf{s}) \rangle$. If the structure factor is separated into the average and fluctuating terms, $F = \langle F \rangle + \tilde{F}$, the total intensity (Equation 10) becomes

$$I(\mathbf{s}) = N^{-1} \langle F(\mathbf{s}) \rangle^2 + N^{-1} \left\langle \tilde{F}(\mathbf{s})^2 \right\rangle. \tag{11}$$

We can further define an average unit-cell electron density, in which the densities of all unit cells are superimposed, $\langle \rho_{\text{cell}}(\mathbf{r}) \rangle$, and corresponding structure factor $\langle F_{\text{cell}}(\mathbf{s}) \rangle$. If the unit cells under consideration are equivalent, ensemble averaging is equivalent to spatial averaging, and the two average structure factors are related as follows:

$$\langle F(\mathbf{s}) \rangle = \langle F_{\text{cell}}(\mathbf{s}) \rangle \sum_{\mathbf{n}} e^{2\pi i \mathbf{s}\cdot\mathbf{r_n}}, \tag{12}$$

where the sum runs over all unit cells, N, and $\mathbf{r_n}$ is the origin of each unit cell. If $\mathbf{s}$ happens to coincide with a node of the reciprocal lattice $\mathbf{g_h}$ ($\mathbf{h}$ is the vector of Miller indices, h, k, and l), the phase factor in the sum is equal to 1 for all $\mathbf{n}$, and the sum evaluates to N. When N is large, the amplitude of the lattice term falls off very quickly with $|\mathbf{s} - \mathbf{g_h}|$, and as $N \to \infty$, it can be written as a sum of delta functions as follows:

$$\lim_{N\to\infty} \left| \sum_{\mathbf{n}} e^{2\pi i \mathbf{s}\cdot\mathbf{r_n}} \right|^2 = N v_c^* \sum_{\mathbf{h}} \delta(\mathbf{s} - \mathbf{g_h}), \tag{13}$$

where v_c^* is the volume of a unit cell of the reciprocal lattice. Thus, for the large crystal, we can combine Equations 11-13 to write the intensity per unit cell as follows:

$$I(\mathbf{s}) = \langle F_{\text{cell}}(\mathbf{s}) \rangle^2 \sum_{h} v_c^* \delta(\mathbf{s} - \mathbf{g_h}) + N^{-1} \left\langle \tilde{F}(\mathbf{s})^2 \right\rangle. \tag{14}$$

The second term of Equation 14 is the diffuse scattering per unit cell,

$$\begin{aligned} I_D(\mathbf{s}) &= N^{-1} \left\langle \tilde{F}(\mathbf{s})^2 \right\rangle \\ &= N^{-1} \left(\left\langle F(\mathbf{s})^2 \right\rangle - \langle F(\mathbf{s}) \rangle^2 \right). \end{aligned} \tag{15}$$

The first term of Equation 14 contains the Bragg peaks. We can define the Bragg intensity as follows:

$$I_B(\mathbf{s}) = \langle F_{\text{cell}}(\mathbf{s}) \rangle^2, \tag{16}$$

which is obtained for $\mathbf{s} = \mathbf{g_h}$ upon integration of the Bragg peaks in reciprocal space. With these definitions (Equations 15-16), the coherent intensity from a macroscopic crystal (Equation 14) is as follows:

$$I(\mathbf{s}) = I_D(\mathbf{s}) + I_B(\mathbf{s}) \sum_{h} v_c^* \delta(\mathbf{s} - \mathbf{g_h}). \tag{17}$$

1.3 Diffuse Patterson function

The diffuse Patterson function, or three-dimensional difference pair distance distribution function (3D-ΔPDF), is defined as the mean autocorrelation of the electron density fluctuations per unit cell, as follows:

$$\begin{aligned}\Delta\mathrm{PDF}(\mathbf{r}) &= N^{-1}\left\langle \Delta\rho\star\Delta\rho\right\rangle(\mathbf{r}) \\ &= N^{-1}\int\left\langle \Delta\rho(\mathbf{u})\Delta\rho(\mathbf{r}+\mathbf{u})\right\rangle d^3\mathbf{u}\,, \end{aligned} \tag{18}$$

where $\Delta\rho = \rho - \langle\rho\rangle$ is the difference between the instantaneous electron density and the ensemble average. The diffuse intensity per unit cell (Equation 15) can be rearranged as:

$$I_D(\mathbf{s}) = N^{-1}\left\langle \left|F(\mathbf{s}) - \langle F(\mathbf{s})\rangle\right|^2\right\rangle. \tag{19}$$

Plugging in the definition of the structure factor (Equation 9), we see that I_D is the Fourier transform of the ΔPDF, as follows:

$$\begin{aligned}I_D(\mathbf{s}) &= N^{-1}\left\langle \left|\int\Delta\rho(\mathbf{r})e^{2\pi i\mathbf{s}\cdot\mathbf{r}}d^3\mathbf{r}\right|^2\right\rangle \\ &= N^{-1}\int\left\langle \Delta\rho\star\Delta\rho\right\rangle(\mathbf{r})\,e^{2\pi i\mathbf{s}\cdot\mathbf{r}}d^3\mathbf{r} \\ &= \int\Delta\mathrm{PDF}(\mathbf{r})\,e^{2\pi i\mathbf{s}\cdot\mathbf{r}}d^3\mathbf{r}. \end{aligned} \tag{20}$$

Thus, I_D and ΔPDF are a Fourier transform pair, and the inverse transform is:

$$\Delta\mathrm{PDF}(\mathbf{r}) = \int I_D(\mathbf{s})\,e^{-2\pi i\mathbf{s}\cdot\mathbf{r}}d^3\mathbf{s}. \tag{21}$$

1.4 Scattering simulation

In order to simulate total scattering without having to model the entire crystal, it is useful to simulate a small number of unit cells and to impose periodic boundary conditions by what is known as the supercell method [13–15]. Given a supercell consisting of a three-dimensional array of $N_{\mathrm{scell}} = N_1\times N_2\times N_3$ unit cells, a "supercell reciprocal lattice" can be defined, which subdivides the regular reciprocal lattice by N_1 along $\mathbf{a}^*$, N_2 along $\mathbf{b}^*$, and N_3 along $\mathbf{c}^*$. The total intensity per unit cell (Equation 10) is calculated in the same manner as usual (Equation 11), except that the structure factor F_{scell} is now a periodic function whose Fourier transform is defined only at nodes of the reciprocal lattice of the supercell. The mean of F_{scell}, evaluated at the Bragg peak locations (the nodes of the unit cell reciprocal lattice, $\mathbf{g_h}$) is proportional to the regular unit cell structure factor F_{cell}:

$$\left|\langle F_{\mathrm{scell}}(\mathbf{s}=\mathbf{g_h})\rangle\right| = N_{\mathrm{scell}}\left|\langle F_{\mathrm{cell}}(\mathbf{s})\rangle\right|. \tag{22}$$

Thus, we can calculate the Bragg intensity per unit cell (Equation 16) from a supercell simulation as follows:

$$I_B(\mathbf{s}) = N_{\mathrm{scell}}^{-2}\left\langle F_{\mathrm{scell}}(\mathbf{s})\right\rangle^2, \tag{23}$$

where F_{scell} is evaluated only at nodes of the regular, unit-cell reciprocal lattice. The diffuse intensity per unit cell is defined in the same way as the macroscopic crystal case (Equation 15), except that it is no longer a continuous function of $\mathbf{s}$ and is evaluated only at the nodes of the supercell reciprocal lattice.

2 Experimental Corrections

Here we consider geometric and experimental corrections that relate the photon counts observed by a detector to the coherent intensity per unit cell, $I(\mathbf{s})$ (see Section 1 and Equation 10). The first section describes the factors depending on the scattering geometry. The second section describes corrections to the Bragg intensity for a crystal that rotates continuously during the exposure. The final section describes a method for placing the total intensity on an absolute scale and subtracting the incoherent contribution.

2.1 Geometric factors

We consider corrections applicable to the geometry normally used in macromolecular crystallography, which consists of a planar X-ray detector with square pixels and a sample that is rotated about a fixed spindle axis. Corrections are derived assuming that detection occurs in the far field from a small sample, so that the illuminated portion of the sample acts as a point-like source.

In Section 1, Equation 8 relates the scattered flux $J(\hat{\mathbf{s}}')$ in the direction $\hat{\mathbf{s}}'$ to the coherent and incoherent intensities. Two prefactors in that equation, the polarization P and solid angle $\Delta\Omega$, depend on the scattering geometry. The solid angle of the pixel can be calculated as follows:

$$\Delta\Omega = (\Delta x \Delta y / d^2) \cos\omega, \tag{24}$$

where d is the distance between the sample and detector pixel, Δx and Δy are the pixel width and height, and ω is the angle between the detector surface normal and the path of the incident photon. The polarization factor depends on the scattering geometry as well as X-ray source properties: the fraction of polarization, p, and the vector normal to the plane of polarization, $\hat{\mathbf{n}}$. In terms of these parameters, the polarization factor can be written as follows [16]:

$$P = p\left(1 + \left(\hat{\mathbf{s}}' \cdot \hat{\mathbf{s}}_0\right)^2\right) + (1-2p)\left(1 - \left(\hat{\mathbf{s}}' \cdot \hat{\mathbf{n}}\right)^2\right). \tag{25}$$

In addition to solid angle and polarization, we must also correct for the efficiency of detection. In an ideal situation, the expected number of photons recorded by a detector pixel, n, would be equal to the scattered flux (Equation 8) times the exposure time: $n = J\Delta t$. However, two non-ideal effects may be important. First is the possibility that the detector pixel does not absorb all of the photons it intercepts. For a pixel of thickness δ and absorption coefficient of the sensor κ, the fraction of photons absorbed, E, is as follows:

$$E = 1 - \exp(-\kappa\delta/\cos\omega). \tag{26}$$

Second is the possibility that the material between the sample and detector absorbs or scatters some of the X-rays before they can be detected. For example, if a uniform material (such as air) with attenuation coefficient μ occupies the space between the sample and the detector (the path length is d), the probability that a photon is transmitted, A, is as follows:

$$A = \exp(-\mu d). \tag{27}$$

The sample rotates during the exposure, so that each pixel samples a range of scattering vectors in the reference frame of the sample. Let $\hat{\mathbf{m}}$ be the spindle axis, ϕ the spindle angle, Δt the exposure time, and $\Delta\phi/\Delta t$ the rate of rotation. Then, for small $\Delta\phi$ and $\Delta\Omega$, the volume element swept by the pixel in reciprocal space is as follows [10]:

$$\Delta v^* = \Delta\phi\Delta\Omega\lambda^{-3}\left|\hat{\mathbf{m}} \cdot \left(\hat{\mathbf{s}} \times \hat{\mathbf{s}}'\right)\right|. \tag{28}$$

The measured intensity thus depends on the average flux (Equation 8) for this volume,

$$\langle J \rangle_{\Delta v^*} = J_0 \Delta\Omega r_e^2 P \left(V_0/v_c\right) \langle I + S \rangle_{\Delta v^*}, \tag{29}$$

where the brackets signify averaging over the volume element Δv^*, as follows:

$$\langle I + S \rangle_{\Delta v^*} \equiv \frac{1}{|\Delta v^*|} \int_{\Delta v^*} d^3\mathbf{s} \left(I(\mathbf{s}) + S(\mathbf{s})\right). \tag{30}$$

Finally, there may be other photons detected during the exposure that come from background sources, such as air scatter. The background scattering rate for each pixel, r_b, will be sample and instrument-dependent, but it can be estimated experimentally, for example by performing a measurement with the sample removed.

With all corrections put together, the expected number of photons detected is related to the intensity per unit cell as follows:

$$n = \Delta t \left(r_b + \Delta\Omega E A P J_0 r_e^2 \left(V_0/v_c\right) \langle I + S \rangle_{\Delta v^*}\right). \tag{31}$$

2.2 Corrections to experimental data

Equation 31 relates the measured photon counts to the signal of interest: the coherent intensity per unit cell, I, which consists of the Bragg and diffuse components. Here we describe the data processing steps that are necessary to convert photon counts into estimates of I_B and I_D on an absolute scale.

In a standard macromolecular crystallography experiment, intensity is determined on an arbitrary scale (this is because the incident flux, the illuminated volume, or both, are typically unknown), and the background scattering is not measured. This is adequate for Bragg intensity determination because the intensity is integrated relative to a local background, and the overall scale factor is fit during model refinement. For diffuse scattering, it is necessary to measure and subtract the background. As a first step in data analysis, the background and geometric corrections are applied to the measured photon counts, so that one obtains an intensity on an arbitrary scale, which we call I_{meas}, as follows:

$$I_{\text{meas}} \equiv \frac{n_{\text{meas}}/\Delta t - r_b}{\Delta\Omega EAP}, \tag{32}$$

where the factors in the denominator are defined in Equations 24-27. This intensity is proportional to true intensity per unit cell, with the expected theoretical relationship (Equations 31and 32):

$$I_{\text{meas}} \propto \langle I + S \rangle_{\Delta v^*}. \tag{33}$$

For a given exposure, the prefactor $\left(J_0 r_e^2 V_0 / v_c\right)$ is a constant that is the same for all pixels.

The next step is to separate I_{meas} into Bragg and continuous scattering components. This is simplest in the case where the coherent intensity I has a diffuse component that varies gradually on the scale of Δv^* and Bragg peaks that are delta function-like on this scale (Equation 17). Then, I_{meas} can be treated differently depending on whether the integration volume Δv^* contains a Bragg peak. If it does not, the intensity can be factored out of the integral (Equation 30), and we have $\langle I + S \rangle_{\Delta v^*} \approx I_D + S$. If the volume contains a Bragg peak (at $\mathbf{s} = \mathbf{g_h}$), we have $\langle I + S \rangle_{\Delta v^*} \approx I_D + S + (v_c^*/\Delta v^*) I_B$. Thus, the Bragg intensity can be estimated from I_{meas} by first subtracting the continuous scattering beneath the Bragg peak and then scaling by $\Delta v^*/v_c^*$ (the Lorentz correction), as follows:

$$I_B \propto (\Delta v^*/v_c^*)\,(I_{\text{meas}} - I_{\text{bkg}}), \tag{34}$$

where I_{bkg} is an estimate for the continuous scattering contribution at $\mathbf{h}$.

2.3 Incoherent scattering and absolute intensity

A convenient method for determining the absolute scale factor based on the integral of the total intensity was first described by Krogh-Moe [17] and further elucidated by Norman [18]. The total intensity includes the coherent scattering (Bragg and diffuse) as well as incoherent Compton scattering. The total integrated intensity (per unit cell) is defined as follows:

$$I_{\text{total}} = \int d^3\mathbf{s}\,\left(I(\mathbf{s}) + S(\mathbf{s})\right), \tag{35}$$

where I and S are the coherent and incoherent scattering per unit cell. Compton scattering is insensitive to molecular structure and can be calculated given the atomic inventory of the unit cell as follows:

$$S = \sum_{\text{n}} S_n(s), \tag{36}$$

where the sum runs over all atoms in the unit cell and $S_n(s)$ are the atomic incoherent scattering functions [11, 12]. Application of Parseval's theorem from harmonic analysis allows us to write the integral of the elastic scattering in terms of independent contributions from each atom, as follows:

$$\int d^3\mathbf{s} I(\mathbf{s}) = \int_0^\infty 4\pi s^2 ds \sum_n f_n^2(s), \tag{37}$$

where f_n are the coherent atomic scattering factors [19]. Thus, both the coherent and incoherent contributions to the total intensity can be calculated from the atomic scattering functions, as follows:

$$I_{\text{total}} = \int_0^\infty 4\pi s^2 ds \sum_n \left(f_n^2(s) + S_n(s)\right). \tag{38}$$

Alternatively, the total intensity can be found by directly integrating the measured three-dimensional reciprocal space map. Care must be taken however to place Bragg and diffuse intensities on the same (arbitrary) scale. If Bragg peaks are modeled as delta functions (Equation 17), the intensity integral is as follows:

$$\begin{aligned} I_{\text{total}} &= \int d^3\mathbf{s}\,[I_D(\mathbf{s}) + I_B(\mathbf{s}) v_c^* \delta(\mathbf{s} - \mathbf{g_h}) + S(\mathbf{s})] \\ &= v_c^* \sum_{\mathbf{h}} I_B(\mathbf{g_h}) + \int d^3\mathbf{s}\,[I_D(\mathbf{s}) + S(\mathbf{s})]. \end{aligned} \tag{39}$$

As discussed in the previous section, the Bragg intensity and the continuous scattering (proportional to $I_D + S$) are measured on an arbitrary scale. To determine the unknown scale factor, the total measured intensity (Equation 39) can be compared with the calculated theoretical intensity (Equation 38). There are two complications, however. The first is that the Bragg peak at **h=0** is unmeasurable. The second is that the limits of integration in Equations 38 and 39 go to infinity, while the measurement ends at some finite maximum scattering vector, s_{max}. To overcome these complications in the theoretical scattering, the **h=0** Bragg intensity is subtracted and the integral truncated at s_{max}, as follows:

$$I_{\text{total, predicted}} = -Z^2 + \int_0^{s_{\text{max}}} 4\pi s^2 ds \sum_n \left(f_n^2(s) + S_n(s)\right), \tag{40}$$

where $Z = \sum_n f_n(0)$ is the number of electrons in the unit cell. This is compared with the integral of the measured data, over the same region of reciprocal space:

$$I_{\text{total, measured}} = v_c^* \sum_{\mathbf{h} \neq 0} I_B(\mathbf{g_h}) + \int_{|s| < s_{\text{max}}} d^3\mathbf{s}\, I_C(\mathbf{s}), \tag{41}$$

where I_B and I_C are the measured Bragg and continuous intensities on an arbitrary scale. The scale factor is calculated from the ratio of predicted and measured total scattering (Equations 40 and 41).

3 Lattice Dynamics Simulation

Lattice dynamics techniques are used to calculate the vibrational frequencies and normal modes (dispersion relations) of atomic or molecular crystals [20, 21], often in the context of diffuse scattering [22, 23]. Here we consider a class of lattice models where the forces between atoms are modeled as a network of springs, and the atoms are assigned to groups that move collectively as rigid bodies.

3.1 Equations of motion in a rigid-body coordinate system

The equations of motion for the network can be expressed using a generalized coordinate system for rigid-body displacement. The instantaneous displacement of a rigid group from its resting position is described by a six-coordinate vector, $\mathbf{w} = (t_1, t_2, t_3, \lambda_1, \lambda_2, \lambda_3)$, where $\mathbf{t}$ and λ are the vectors of translation and libration [6]. For small rotations, the displacement $\mathbf{u}$ of an atom in the group is related to $\mathbf{w}$ by a linear operator: $\mathbf{u} = \mathbf{A}(\mathbf{r})\mathbf{w}$, where $\mathbf{A}$ depends on the coordinates of the atom at rest, $\mathbf{r} = (r_1, r_2, r_3)$, as follows:

$$\mathbf{A}(\mathbf{r}) = \begin{bmatrix} 1 & 0 & 0 & 0 & r_3 & -r_2 \\ 0 & 1 & 0 & -r_3 & 0 & r_1 \\ 0 & 0 & 1 & r_2 & -r_1 & 0 \end{bmatrix}. \tag{42}$$

In the derivation below, we introduce a compact notation where the cartesian displacements of all n atoms in a unit cell l are represented by a $3n$-dimensional column vector $\mathbf{u}_{(l)}$, and similarly the generalized coordinates of all m groups in the unit cell are represented by a $6m$-dimensional column vector $\mathbf{w}_{(l)}$. A $3n$-by-$6m$ matrix $\mathbf{A}$ is constructed so that

$$\mathbf{u}_{(l)} = \mathbf{A}\,\mathbf{w}_{(l)}. \tag{43}$$

Using this notation, the harmonic part of the total potential energy is

$$V^{(2)} = \frac{1}{2}\sum_{l,l'} \mathbf{w}_{(l)}^{\mathrm{T}} \mathbf{V}_{(l,l')} \mathbf{w}_{(l')}, \tag{44}$$

where $\mathbf{V}_{(l,l')}$ is the portion of the Hessian matrix (or the force constants matrix) pertaining to the interaction between a unit cell l and l', defined as follows:

$$\mathbf{V}_{(l,l')} = \mathbf{A}^{\mathrm{T}} \left. \frac{\partial^2 V}{\partial \mathbf{u}_{(l)}\, \partial \mathbf{u}_{(l')}^{\mathrm{T}}} \right|_{\mathbf{u}=0} \mathbf{A}, \tag{45}$$

where V is the total potential energy. The total kinetic energy is

$$T = \frac{1}{2}\sum_{l} \dot{\mathbf{w}}_{(l)}^{\mathrm{T}} \mathbf{M} \dot{\mathbf{w}}_{(l)}, \tag{46}$$

where $\mathbf{M}$ is the matrix of generalized masses (which includes the moments of inertia), defined as follows:

$$\mathbf{M} = \mathbf{A}^{\mathrm{T}} \left(\mathrm{diag}(\mathbf{m}) \otimes \mathbf{I}_3\right) \mathbf{A}, \tag{47}$$

where $\mathrm{diag}(\mathbf{m})$ is a square matrix with the atomic masses along the diagonal, $\mathbf{I}_3$ is the 3×3 identity matrix, and $\otimes$ is the Kronecker product.

The equations of motion can be derived from the kinetic and potential energies (Equations 44 and 46) using Lagrangian mechanics, with the following result:

$$\mathbf{M}\ddot{\mathbf{w}}_{(l)} = -\sum_{l'} \mathbf{V}_{(l,l')} \mathbf{w}_{(l')}. \tag{48}$$

The generalized mass matrix is positive definite and can be decomposed as $\mathbf{M} = \mathbf{L}\mathbf{L}^{\mathrm{T}}$, where $\mathbf{L}$ is a lower triangular matrix (found by Cholesky decomposition).

3.2 Normal modes

The Born / von-Karman approach [20–22] can be used to solve the equations of motion (Equation 48) by transforming from real space to reciprocal space ($\mathbf{k}$-space, where $\mathbf{k}$ is the wavevector). First, displacement-wave solutions are proposed with the following form:

$$\mathbf{w}_{(l)}^{\pm} = \mathbf{L}^{-\mathrm{T}} \mathbf{e} \exp\left[i\left(\mathbf{k}\cdot\mathbf{r}_l \pm \omega t\right)\right], \tag{49}$$

where $\mathbf{e}$ is a (complex) polarization vector, $\mathbf{r}_l$ is the origin of unit cell l, ω is the angular frequency, and t is time. Substituting these solutions into the equations of motion,

$$\omega^2 \mathbf{e} = \left\{ \mathbf{L}^{-1} \sum_{l'} \mathbf{V}_{(l,l')} \exp\left[i\mathbf{k}\cdot(\mathbf{r}_{l'} - \mathbf{r}_l)\right] \mathbf{L}^{-\mathrm{T}} \right\} \mathbf{e}. \tag{50}$$

This can be cast in the form of an eigenvalue equation, $\omega^2 \mathbf{e} = \mathbf{D}_{(\mathbf{k})}\mathbf{e}$, where $\mathbf{D}_{(\mathbf{k})}$ is known as the dynamical matrix. Let $\omega_{(\mathbf{k},j)}^2$ and $\mathbf{e}_{(\mathbf{k},j)}$ be the jth eigenvalue and eigenvector of $\mathbf{D}_{(\mathbf{k})}$. These satisfy

$$\omega_{(\mathbf{k},j)}^2 \mathbf{e}_{(\mathbf{k},j)} = \mathbf{D}_{(\mathbf{k})} \mathbf{e}_{(\mathbf{k},j)}. \tag{51}$$

Here we consider finite supercells consisting of N primitive unit cells and periodic boundary conditions so that $\mathbf{k}$ is restricted to a discrete set. It can be shown [21] that the general solution is a sum of all allowed waves, or normal modes, as follows:

$$\mathbf{w}_{(l)} = \sum_{\mathbf{k}} \sum_{j} \sigma_{(\mathbf{k},j)} \mathbf{L}^{-\mathrm{T}} \mathbf{e}_{(\mathbf{k},j)} \exp\left(i\mathbf{k} \cdot \mathbf{r}_l\right), \tag{52}$$

where $\sigma_{(\mathbf{k},j)}$ are complex, time-dependent amplitudes of the normal modes.

3.3 Displacement covariances for thermally-excited vibrations

In thermodynamic equilibrium, the mean-squared amplitudes of the normal coordinates can be found by applying the equipartition theorem (high-temperature limit):

$$\left\langle \left|\sigma_{(\mathbf{k},j)}\right|^2 \right\rangle = N^{-1} k_B T \omega_{(\mathbf{k},j)}^{-2}. \tag{53}$$

Using this result, the covariance matrix can be derived for the generalized coordinates. It can be expressed in the following compact form:

$$\left\langle \mathbf{w}_{(l)} \mathbf{w}_{(l')}^{\mathrm{T}} \right\rangle = N^{-1} k_B T \sum_{\mathbf{k}} \exp\left(i\mathbf{k} \cdot (\mathbf{r}_l - \mathbf{r}_{l'})\right) \mathbf{K}_{(\mathbf{k})}^{+}, \tag{54}$$

where $\mathbf{K}_{(\mathbf{k})}$ is the Fourier transform of the force constants matrix ($\mathbf{K}_{(\mathbf{k})} = \mathbf{L}\mathbf{D}_{(\mathbf{k})}\mathbf{L}^T$) and $\mathbf{K}_{(\mathbf{k})}^{+}$ is its generalized inverse (in the generalized inverse, normal modes corresponding to rotation and translations of the entire crystal have $\omega = 0$ and are excluded). The covariance of individual atomic displacements can be calculated by projection (Equation 42), as follows:

$$\left\langle \mathbf{u}_{(jl)} \mathbf{u}_{(j'l')}^{\mathrm{T}} \right\rangle = \mathbf{A}(\mathbf{r}_j) \left\langle \mathbf{w}_{(\kappa l)} \mathbf{w}_{(\kappa' l')}^{\mathrm{T}} \right\rangle \mathbf{A}(\mathbf{r}_j)^{\mathrm{T}}, \tag{55}$$

where the index κ refers to the rigid group to which atom j belongs ($\mathbf{w}_{(\kappa l)}$ is a 6-element vector). The self-terms of the covariance matrix are the familiar atomic displacement parameters (ADPs) in protein crystallography:

$$\mathbf{U}_j = \left\langle \mathbf{u}_{(j)} \mathbf{u}_{(j)}^{\mathrm{T}} \right\rangle. \tag{56}$$

The equivalent isotropic B-factor is defined as

$$B_{\mathrm{eq}} = \left(8\pi^2/3\right) \mathrm{tr}\left(\mathbf{U}\right). \tag{57}$$

Because the generalized coordinates of the lattice model $\mathbf{w}$ are the same as those used in TLS refinement [6], the $\mathbf{T}$, $\mathbf{L}$, and $\mathbf{S}$ matrices are 3×3 blocks within the larger covariance matrix for $\mathbf{w}$. For a particular rigid group κ, the matrices are related as follows:

$$\left\langle \mathbf{w}_{(\kappa)} \mathbf{w}_{(\kappa)}^{\mathrm{T}} \right\rangle = \begin{bmatrix} \mathbf{T} & \mathbf{S}^{\mathrm{T}} \\ \mathbf{S} & \mathbf{L} \end{bmatrix}. \tag{58}$$

3.4 Diffuse scattering and the one-phonon approximation

It is customary in phonon scattering theory to represent the reciprocal space coordinate by the momentum transfer vector $\mathbf{q}$ rather than the scattering vector $\mathbf{s}$, with the relationship $\mathbf{q} = 2\pi\mathbf{s}$. We follow this convention below. Since the lattice vibrations are harmonic, the diffuse scattering intensity per unit cell can be calculated in the harmonic approximation:

$$I_D = N^{-1} \sum_{l,l'} e^{i\mathbf{q}\cdot(\mathbf{r}_l - \mathbf{r}_{l'})} \sum_{j,j'} f_j f_{j'} e^{i\mathbf{q}\cdot\left(\mathbf{r}_j - \mathbf{r}_{j'}\right)} T_j T_{j'} \left[T_{lj,l'j'} - 1\right]. \tag{59}$$

The first summation is over all pairs of unit cells indexed by l and l', and $\mathbf{r}_l$ is the origin of unit cell l. The second summation is over all atom pairs in unit cell l (indexed by j) and l' (indexed by j'). Here, f_j is the atomic scattering

factor and $\mathbf{r}_j$ is the average position of the atom relative to the unit cell origin. The factors T_j (the Debye-Waller factor) and $T_{lj,l'j'}$ depend on the self- and cross-terms of the displacement covariance matrix, as follows:

$$T_j = \exp\left[-\frac{1}{2}\mathbf{q}^{\mathrm{T}}\mathbf{U}_j\mathbf{q}\right] \tag{60}$$

and

$$T_{lj,l'j'} = \exp\left[\frac{1}{2}\mathbf{q}^{\mathrm{T}}\left(\left\langle \mathbf{u}_{j'l'}\mathbf{u}_{jl}^{\mathrm{T}}\right\rangle + \left\langle \mathbf{u}_{jl}\mathbf{u}_{j'l'}^{\mathrm{T}}\right\rangle\right)\mathbf{q}\right], \tag{61}$$

where $\mathbf{u}_{jl}$ is the instantaneous displacement of atom j in unit cell l and $\mathbf{U}_j$ is defined in Equation 56.

When the cross-terms of the covariance matrix are small, Equation 61 can be expanded as a Taylor series, and the intensity separated into terms of increasing order ($I = I^{(1)} + I^{(2)} + \ldots$.) where $I^{(1)}$ is the "one-phonon" diffuse scattering, $I^{(2)}$ is the "two-phonon" diffuse scattering, and so on. In the "one-phonon" term, a phonon with wavevector $\mathbf{k}$ contributes only to points in reciprocal space that are displaced by $\mathbf{k}$ from the Bragg peak locations $\mathbf{g_h}$, as follows:

$$I^{(1)}(\mathbf{q} = \mathbf{g_h} - \mathbf{k}) = \sum_n \frac{k_B T}{\omega^2_{(\mathbf{k},n)}} \left| \sum_j f_j T_j \exp\left[i\mathbf{q}\cdot\mathbf{r}_j\right] \mathbf{q}^{\mathrm{T}}\mathbf{A}(\mathbf{r}_j)\mathbf{L}^{-\mathrm{T}}_{(\kappa)}\mathbf{e}_{(\kappa,\mathbf{k},n)} \right|^2. \tag{62}$$

This can be written in a more compact form that is computationally convenient by defining the vector-valued "one-phonon structure factor",

$$\mathbf{G}(\kappa, \mathbf{q}) = \mathbf{q}^{\mathrm{T}} \sum_j f_j T_j \exp\left[i\mathbf{q}\cdot\mathbf{r}_j\right] \mathbf{A}(\mathbf{r}_j). \tag{63}$$

Then, the one-phonon intensity can be written as follows:

$$I^{(1)}(\mathbf{q} = \mathbf{g_h} - \mathbf{k}) = k_B T\, \mathbf{G}(\mathbf{q})\mathbf{K}^{+}_{(\mathbf{k})}\mathbf{G}^{\dagger}(\mathbf{q}), \tag{64}$$

where $\mathbf{K}^{+}_{(\mathbf{k})}$ is the generalized inverse of the force constants matrix (see Equation 54).

Supplementary References

1. Ayyer, K. *et al.* Macromolecular diffractive imaging using imperfect crystals. *Nature* **530,** 202–206 (2016).
2. Kabsch, W. Integration, scaling, space-group assignment and post-refinement. *Acta Crystallogr., Sect. D: Biol. Crystallogr.* **66,** 133–144 (2010).
3. Evans, P. R. & Murshudov, G. N. How good are my data and what is the resolution? *Acta Crystallogr., Sect. D: Biol. Crystallogr.* **69,** 1204–1214 (2013).
4. Chen, V. B. *et al.* MolProbity: all-atom structure validation for macromolecular crystallography. *Acta Crystallogr., Sect. D: Biol. Crystallogr.* **66,** 12–21 (2010).
5. Meinhold, L., Merzel, F. & Smith, J. C. Lattice Dynamics of a Protein Crystal. *Phys. Rev. Lett.* **99,** 138101 (2007).
6. Schomaker, V. & Trueblood, K. N. On the rigid-body motion of molecules in crystals. *Acta Crystallogr., Sect. B: Struct. Crystallogr. Cryst. Chem.* **24,** 63–76 (1968).
7. Riccardi, D., Cui, Q. & Phillips Jr., G. N. Evaluating Elastic Network Models of Crystalline Biological Molecules with Temperature Factors, Correlated Motions, and Diffuse X-Ray Scattering. *Biophys. J.* **99,** 2616–2625 (2010).
8. Benschoten, A. H. V. *et al.* Measuring and modeling diffuse scattering in protein X-ray crystallography. *Proc. Natl. Acad. Sci. U. S. A.* **113,** 4069–4074 (2016).
9. Meisburger, S. P., Thomas, W. C., Watkins, M. B. & Ando, N. X-ray Scattering Studies of Protein Structural Dynamics. *Chem. Rev.* **117,** 7615–72 (2017).
10. Als-Nielsen, J. & McMorrow, D. *Elements of Modern X-ray Physics* (John Wiley & Sons, 2011).
11. Alexandropoulos, N. G., Cooper, M. J., Suortti, P. & Willis, B. T. M. in *International Tables for Crystallography Volume C: Mathematical, physical and chemical tables* (ed Prince, E.) 653–665 (Springer Netherlands, Dordrecht, 2004).
12. Hubbell, J. H. *et al.* Atomic form factors, incoherent scattering functions, and photon scattering cross sections. *J. Phys. Chem. Ref. Data* **4,** 471–538 (1975).
13. Nield, V. M., Keen, D. A. & McGreevy, R. L. The interpretation of single-crystal diffuse scattering using reverse Monte Carlo modelling. *Acta Crystallogr., Sect. A: Found. Crystallogr.* **51,** 763–771 (1995).
14. Janowski, P. A., Liu, C., Deckman, J. & Case, D. A. Molecular dynamics simulation of triclinic lysozyme in a crystal lattice. *Protein Sci.* **25,** 87–102 (2016).
15. Wall, M. E. Internal protein motions in molecular-dynamics simulations of Bragg and diffuse X-ray scattering. *IUCrJ* **5,** 172–181 (2018).
16. Kabsch, W. Evaluation of single-crystal X-ray diffraction data from a position-sensitive detector. *J. Appl. Cryst.* **21,** 916–924 (1988).
17. Krogh-Moe, J. A method for converting experimental X-ray intensities to an absolute scale. *Acta Crystallogr.* **9,** 951–953 (1956).
18. Norman, N. The Fourier transform method for normalizing intensities. *Acta Crystallogr.* **10,** 370–373 (1957).
19. Brown, P. J., Fox, A. G., Maslen, E. N., O'Keefe, M. A. & Willis, B. T. M. in *International Tables for Crystallography Volume C* (ed Prince, E.) 554–595 (Springer, 2006).
20. Born, M. & Huang, K. *Dynamical theory of crystal lattices* (Clarendon Press, 1954).
21. Eckold, G. in *International Tables for Crystallography Volume D: Physical properties of crystals* (ed Authier, A.) 266–293 (Springer Netherlands, Dordrecht, 2003).
22. Willis, B. T. M. in *International Tables for Crystallography Volume B: Reciprocal space* (ed Shmueli, U.) 400–406 (Springer Netherlands, Dordrecht, 2001).

23. Welberry, T. R. & Weber, T. One hundred years of diffuse scattering. *Crystallogr. Rev.* **22,** 2–78 (2016).
