# Supplementary material for: Diffuse X-ray scattering from correlated motions in a protein crystal
Source: Nat Commun. 2020 Mar 9;11:1271. doi: 10.1038/s41467-020-14933-6 (PMC7062842; doi:10.1038/s41467-020-14933-6)
Supplement: Supplementary file 3 — Description of Additional Supplementary Files [file 41467_2020_14933_MOESM3_ESM.pdf]

Description of Additional Supplementary Files

File Name: Supplementary Movie 1

Description: Variational diffuse intensity in the **a*-b*** plane (constant Miller index *l*, shown in lower right). The intensity scale is the same as in Fig. 1C in the Main Text.

File Name: Supplementary Movie 2

Description: Acoustic vibrations of triclinic lysozyme according to the lattice dynamics model refined using diffuse scattering. The animation shows three acoustic modes with different polarization directions, $\hat{\mathbf{p}}$ (two polarized mostly transverse and one longitudinal) for the wavevector $\mathbf{k} = \mathbf{a}^*/13$ (the Γ-M direction in Supplementary Table 3 and Supplementary Fig. 15). To aid in visualizing the small motions, the vibrational amplitudes were multiplied by 100 and the frequencies were set to $\nu \sim 1 s^{-1}$.
